# Silicone Elastomer Composites Fabricated with MgO and MgO-Multi-Wall Carbon Nanotubes with Improved Thermal Conductivity

**DOI:** 10.3390/nano11123418

**Published:** 2021-12-16

**Authors:** Christopher Kagenda, Jae Wook Lee, Fida Hussain Memon, Faheem Ahmed, Anupama Samantasinghar, Muhammad Wasim Akhtar, Abdul Khalique, Kyung Hyun Choi

**Affiliations:** 1 Faculty of Science, Faculty of Chemistry, Chemical Engineering, Kyambogo University, Kampala P.O. Box 1, Uganda; kagztopher@gmail.com; 2 School of Semiconductor and Chemical Engineering, Chonbuk National University, Jeonju-si 54896, Korea; 3 Advanced Micro Mechatronics Labortory, Department of Mechatronics Engineering, Jeju National University, Jeju-si 63243, Korea; jaewook482@gmail.com (J.W.L.); fida.hussain@iba-suk.edu.pk (F.H.M.); faheeemahmedlangah@gmail.com (F.A.); anupamasamantasinghar31@gmail.com (A.S.); 4 Department of Electrical Engineering, Sukkur IBA University, Sukkur 65200, Pakistan; 5 Department of Metallurgy and Materials Engineering, Mehran University of Engineering & Technology, Jamshoro 76062, Pakistan; danialmemon79@gmail.com

**Keywords:** thermal conductivity, silicone elastomer, multi-wall carbon nanotubes

## Abstract

The effect of multiwall carbon nanotubes (MWCNTs) and magnesium oxide (MgO) on the thermal conductivity of MWCNTs and MgO-reinforced silicone rubber was studied. The increment of thermal conductivity was found to be linear with respect to increased loading of MgO. In order to improve the thermal transportation of phonons 0.3 wt % and 0.5 wt % of MWCNTs were added as filler to MgO-reinforced silicone rubber. The MWCNTs were functionalized by hydrogen peroxide (H_2_O_2_) to activate organic groups onto the surface of MWCNTs. These functional groups improved the compatibility and adhesion and act as bridging agents between MWCNTs and silicone elastomer, resulting in the formation of active conductive pathways between MgO and MWCNTs in the silicone elastomer. The surface functionalization was confirmed with XRD and FTIR spectroscopy. Raman spectroscopy confirms the pristine structure of MWCNTs after oxidation with H_2_O_2_. The thermal conductivity is improved to 1 W/m·K with the addition of 20 vol% with 0.5 wt % of MWCNTs, which is an ~8-fold increment in comparison to neat elastomer. Improved thermal conductive properties of MgO-MWCNTs elastomer composite will be a potential replacement for conventional thermal interface materials.

## 1. Introduction

Due to their light weight, high dimensional accuracy, and adequate properties, polymeric materials have gained significant importance in recent times. Use of polymeric materials has exponentially increased and polymers can extensively be used in fabrication of composite materials. Due to their promising insulating qualities, good thermal stability, high strength, sufficient mechanical properties, biocompatibility, and simplicity in fabrication, elastomers are the second most widely used thermoset polymers after polyester [1,2,3,4,5,6,7,8,9]. Besides these properties, there are few properties like thermal conductivity that are quite low and do not meet the requirements of many applications. It is important to address these issues to deal with the demand for thermal management applications. The miniaturizing in electronic components makes the heat dissipation problem more significant and drastic. A fractional rise in temperature has consequent effects on the reliability and lifespan of electronic components [10,11]. Thermal interface materials are employed to occupy air gaps between the assembly when solid parts are joined together. Increase in transportation of phonons by reducing the contact resistance. The reliability and performance of modern electronic devices greatly depend on high thermal conductivity of the thermal interface materials [12].

Polymer nanocomposites are alternatives to conventional thermal interface materials. Thermal grease is often used for thermal interface materials, but it is associated with the ‘pump-out’ problem; furthermore, the thickness constraint in phase change materials limits its use for heat dissipation applications. Recently, polymer-based thermally conductive composites are typically made by directly combining a polymer matrix with extremely thermally conductive fillers such as carbon black [13], graphene [14,15], boron niride [16], and alumina [17]; however, the random distribution of fillers in bulk composites typically results in high interfacial thermal resistance and limits the through-plane thermal conductivity in between 1–5 Wm^−1^K^−1^. Modification of filler is one of the probable ways to augment the distribution and interconnective networks among matrix and fillers, thus improving thermal attributes of the composites. The resistance in phonon transportation is curtailed by the formation of strong interfacial connection with filler and matrix by employing functionalization of filler or matrix. Common inorganic fillers—such as alumina, aluminum nitride, and boron nitride—are being employed as thermal conductors for incorporation in polymeric materials [18,19,20].

Magnesium oxide (MgO) is another filler with attractive characteristics. Bulk thermal conductivity of MgO is far better in comparison to other inorganic fillers. MgO is also widely used in polymer molding as a dielectric powder filler. MgO also possesses a low hardness value compared to other inorganic fillers. The deformation behavior of MgO is similar to metals because of its low hardness [21]. The use of MgO as an effective filler for enhancement of thermal properties of the high viscous elastomers is not extensively studied. Thermal conductivity of MgO/polymer composites has focused on matrices with fewer viscosities like epoxy [22]. However, a higher quantity of MgO filler is essential to enhance thermal properties, since this higher quantity deteriorates mechanical properties. Furthermore, it leads towards the non-homogeneous dispersion of filler which causes interstitial gaps between filler matrix which is an important reason for low conductivity even with a high proportion of MgO filler [23]. As a result, thermally conductive films with relatively low filler loading are preferred. A sufficient thermal conductivity value can even be achieved with low filler loading with good thermal contact. Good interference between particle and matrix decreases the thermal resistance and increases heat dissipation [24].

One possible route to escalate the interference in the filler and matrix is by the addition of functional moieties that generate an effective thermal conductive path between the filler particles and the polymer matrix [2,25,26]. Surface modification helps to generate good interfacial bonds and create an effective thermal bridge for the transportation of phonons [27]. Recently, many reports published that the importance of surface modification is evident in the enhancement of thermal conductivity [2,28,29,30,31]. The high aspect ratio and good interface through surface modification are usually key to improve thermal conductivity. Previous reports claim the use of hybrid fillers are effective to enhance thermal conductivity of the composites. [32]. CNTs are intensively used to improve the thermal properties of the composites. The surface modification of CNT with COOH is reported high thermal conductivity [33]. The functional moieties enhance the phonon transportation and thermal conductivity of carbon nanotube fluid as heat transmission channel. The functionalization of CNTs improve the dispersion of the filler in the polymer matrix that counter the agglomeration problem and provide highly thermal conductive path [34]. Another report suggested that the surface modification of graphene with O-phenylenediamine (OPD) aids in augmenting the thermal conductivity of the composite. An increment of 13-fold in in-plane and 4-fold in through-plane thermal conductivity was recorded after the functionalization of graphene [35]. However, the use of a single thermal conductive filler is not that effective and requires a large proportion to achieve adequate thermal conductive characteristics. The use of multi-structured hybrid filler is advantageous to utilize the properties of various fillers which are not possible in the case of single thermal conductive filler [36]. Hetero-structured fillers have highly thermal conductive networks that provide resistance-free transportation of phonons and synergistic improvement in thermal properties [37,38].

Herein, we report a novel approach by incorporating MgO and MWCNT in the matrix of silicon rubber (KE-12). The surface oxidation of MWCNT incorporates oxygen-containing functional groups onto the graphitic surface of CNTs enhances interfacial adhesion as well as establishing a bond with MgO. Furthermore, the high aspect ratio of CNTs helps in generation efficient thermal conductive networks in hybrid structure that decreases the Kapitza resistance and improves pho-non transportation, resulting in improved thermal conductivity of the composite that can be used as potential thermal interface materials (TIMs).

## 2. Experimental Methods

### 2.1. Materials

MWCNTs of high purity were acquired from NanoTech Co. Ltd., Jeonju, South Korea with external diameters of 8–15 nm, inside diameters of 3–5 nm, and lengths of 10–50 µm. Sodium hydroxide (NaOH) and magnesium chloride (MgCl_2_·6H_2_O) were bought from Samchun chemicals Co. Ltd., Jeonju, South Korea. Urea and Ethanol were obtained from DaeJung Chemicals, Jeonju, South Korea. 30% H_2_O_2_ was purchased from Junsei Chemical Co. Ltd., Japan. The RTV silicon rubber KE-12 and its hardener were obtained from Shin-Etsu, Japan. All chemical reagents used in this experiment were of analytical grade and used as received.

### 2.2. Synthesis of MgO

Synthesis of nanoscale MgO was done by mixing 8 g of NaOH with 25 mL of deionized water. Besides, MgCl_2_ solution of (4.0 M) was further added within span of 10 min. Later prepared mixture of solution was stirred using a mechanical stirrer at 60 °C for 1 h. Obtained solution was subsequently aged for 10 h at 60 °C. Synthesized percipitates of MgO were rinsed in ethanol and vacuum dried at 60 °C, with subsequent calcination at 150 °C for 2 h.

### 2.3. Preparation of H_2_O_2_ Surface Modified MWCNTs

0.5 g in 30% H_2_O_2_ of 10 mL MWCNTs were oxidized through stirring for 24 h at the temperature of 65 °C as reported [39]. The oxidized MWCNTs were washed with de-ionized water, using filter paper of 0.45 µm Millipore membrane. The filtrate was dried overnight at the temperature of 110 °C, later vacuum-dried for 6 h at the temperature of 150 °C to expel adsorbed H_2_O_2_ and coupled peroxide functional groups from the surface of the MWCNTs.

### 2.4. Fabrication of Filler/Silicone Elastomer Composites

The same procedure was followed in the fabrication of filler/silicone elastomer composites of MgO and MgO-MWCNTS composites. In MgO/silicone elastomer composites, the filler loading into the elastomer matrix varied from 10 to 40 vol% while in MgO-MWCNTs hybrid/silicone elastomer composite, the MgO loading was maintained at 20 vol% and the amount MWCNTs added were 0.3 and 0.5 vol% to each loading of MgO. In a typical procedure, after the contents were measured in the required amount, they were mixed with the measured amount of silicone resins using a high-speed Thinky mixer at the speed of 1500 RPM for 15 min. Later, hardener was added to the mixture, which was subsequently casted into furnished molds, followed by curing at ambient temperature for 48 h.

## 3. Results and Discussion

### 3.1. Macroscopic and Structural Evolution MgO Nanoparticles

**Figure 1:**
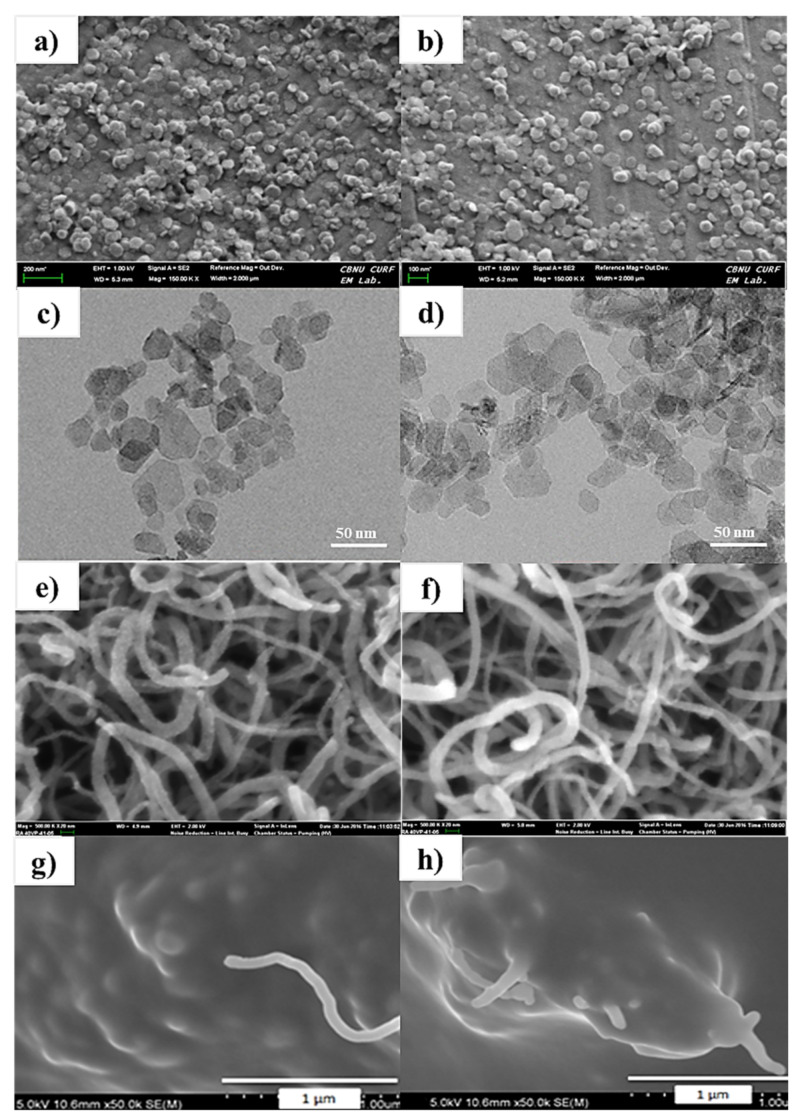
FE-SEM images of (**a**) Mg(OH)_2_ and (**b**) MgO; HR-TEM images of urea assisted (planet-like) (**c**) Mg(OH)_2_ and (**d**) MgO, morphology of MWCNTs; FESEM image of (**e**) controlled MWCNTs; (**f**) H_2_O_2_ treated MWCNTs, FE-SEM images of cross-section of MgO-MWCNT/silicone resins composites; (**g**) 20 vol%–0.5 vol% H_2_O_2_-MWCNTs MWCNTs; (**h**) 30 vol%–0.5 vol% H_2_O_2_-MWCNTs.

The surface topography of the prepared Mg (OH)_2_ and MgO nanoparticles was done through FE-SEM and TEM as shown in Figure 1a–d. It is evident from SEM and TEM that the particle size of Mg (OH)_2_ and MgO nanoparticles lies in the range of 50–70 nm before and after calcination as shown in Figure 1. It is observed from the surface morphology of MgO nanoparticles that the basic hexagonal crystalline template of MgO is almost retained after calcination. This insignificant contrast demonstrates that calcination has little effect on the morphology of the nanoparticles. The agglomeration in the particles is due to the presence of the surfactant that was already reported elsewhere [40].

**Figure 2:**
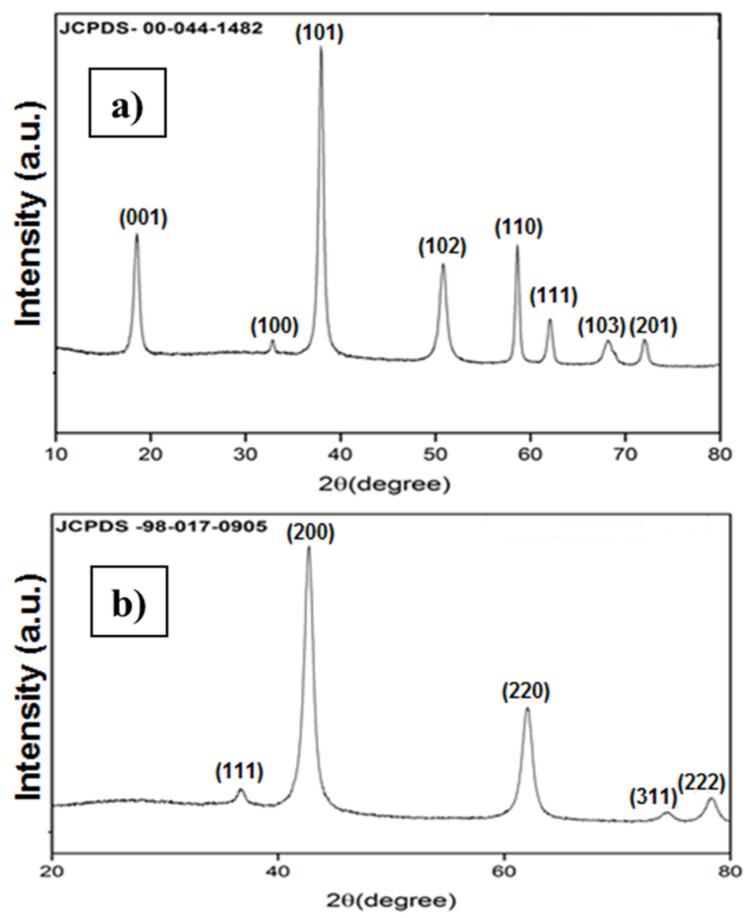
XRD patterns of (**a**) Mg(OH)_2_ and (**b**) MgO.

The crystallinity of Mg(OH)_2_ and MgO was characterized using the XRD spectroscopy as in Figure 2. In Figure 2a, the diffraction lines confirm with the JCPDS card analyzer for Mg(OH)_2_. The XRD spectra of MgO calcinied at 450 °C. The presence of peaks at 2*θ* values of 36.9°, 42.9°, 62.3°, and 74.6° and 78.6° can be indexed to the (1 1 1), (2 0 0), (2 2 0), (3 1 1), and (2 2 2) planes of the face-centered cubic (FCC) structured MgO nanoparticle. The absence of other peaks in the XRD spectra confirms the high crystallinity and purity of MgO nanoparticles (JCPDS file no. 98-17-0905) as shown in (Figure 2b). Average crystallite size was obtained in range of 60 nm, that was calculated using Scherrer’s Equation.

$$D=\frac{K\;\lambda }{\beta \;\cos\Theta }$$

- D= Mean size of the ordered (crystalline) domains;
- K= Constant;
- λ= X-ray wavelength;
- β= Line broadening at half the maximum intensity (FWHM);
- Θ= Peak position

### 3.2. Surface Treatment of Multi-Walled Carbon Nanotubes (MWCNTs)

**Figure 3:**
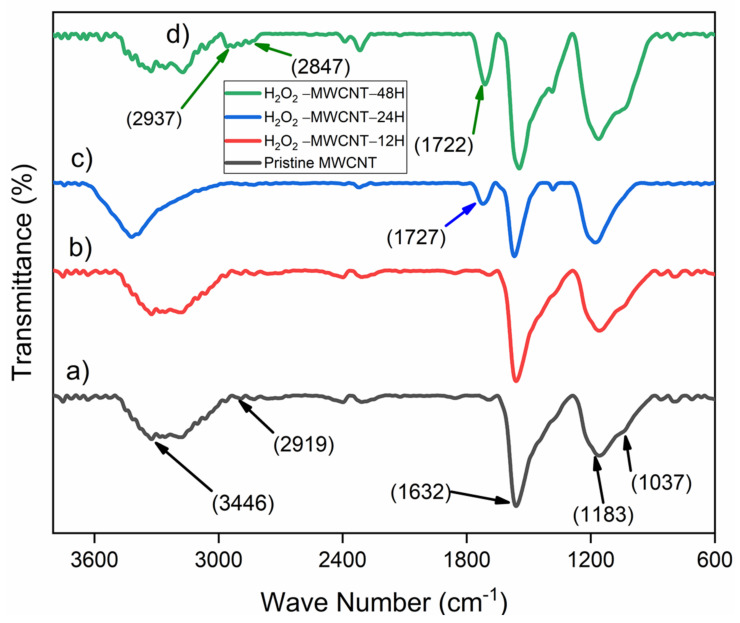
FTIR spectra of MWCNTs and H_2_O_2_-treated MWCNTs. (a) Pristine MWCNTs; (b) H_2_O_2_-MWCNTs for 12 h; (c) H_2_O_2_-MWCNTs for 24 h; and (d) H_2_O_2_-MWCNTs for 48 h.

The surface modification of MWCNT was studied by FTIR spectroscopy. In the pristine MWCNTs spectrum (Figure 3a), pristine MWCNT samples show strong peak around 1632 cm^−1^ attributed to C=C stretching mode. The peak at 1037 cm^−1^ is due to stretching mode of C-O (COH). The peak at 2919 cm^−1^ is due to the asymmetric C–H_n_ and symmetric C–H_n_ stretching mode. The peak at around 3446 cm^−1^ corresponding to OH peak is related to absorbed water molecules on the carbon in the MWCNTs. The results are in good agreement with previously reported literature [41]. H_2_O_2_-treated MWCNTs at 12 h showed the same effect on band peaks as the pristine MWCNTs showed and no oxidation was observed within 12 h of treatment (Figure 3b). However, as the oxidation time is increased, the spectrum (Figure 3c,d) of the oxidized sample shows, the –COOH, –C=O, and –OH band peaks prominently declined. A new peak at 1727 cm^−1^ is appeared due to C=O stretching frequency, that shows the presence of carboxylic group, formed during the oxidation of MWCNTs. After 48 h, the intensity of C=O implies more number of carboxylic groups attached to MWCNTs due to further oxidation. Furthermore, two new peaks are also observed at around 2936 and 2847 cm^−1^ belonging to CH_2_ group on the surface of H_2_O_2_-MWCNT-48 CH shows the higher stability of oxidized MWCNTs in comparison to pristine MWCNTs [42].

**Figure 4:**
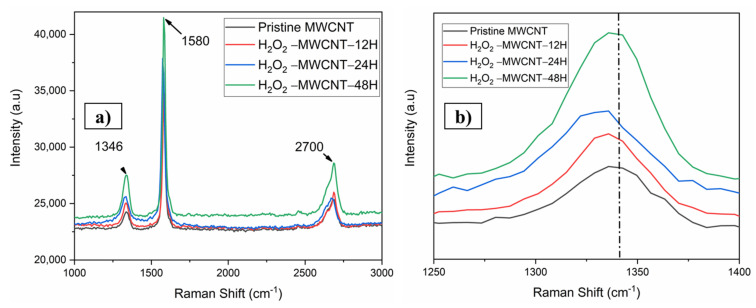
(**a**) Raman spectra of pristine MWCNTs and H_2_O_2_ treated MWCNTs, and (**b**) magnified spectra of D peak.

The Raman spectrum was observed from the MWCNTs (CO-based) at ambient temperature as in Figure 4. Raman spectra exhibits two Raman peaks, namely G(graphite)- and D(disorder)- the band of MWCNTs. The peak at around 1580 cm^−1^ because of the Raman-active *E*_2_g mode analogous to that of graphite [43,44], and the D-peak is because of the breathing modes of the sp^2^ atoms, providing the defects at the first Brillouin zone [45]. The I_G_/I_D_ ratio of the neat MWCNT specimen was 0.86. It is a common indicator of a significant number of structural flaws in MWCNT graphitic structure. Such a structure provides many active sites for more modification as reported [39]. It can also be observed from (Figure 4a) that the vibrational properties of the MWCNT altered the I_G_/I_D_ ratio to 0.85, 0.82, and 0.80 for 12-, 24-, and 48-h treatment with H_2_O_2_ respectively. This I_G_/I_D_ ratio change in the oxidized material has been reported before. It has been associated with the inception of latest defects, and geometrical alterations in MWCNT [39,46,47,48]. More similar observations include the D-band region upshifts in the oxidized sample. The increment of the D peak can be observed which confirms the attachment of functional moieties on the surface of MWCNTs Figure 4b.

FESEM images of pristine MWCNTs and the oxidized MWCNTs (24 h) as shown in Figure 1e,f. The graphitic structure of pristine MWCNTs was very smooth and highly visible in comparisson to oxidized MWCNTs (Figure 1e). After oxidation, the MWCNTs bundles appear exfoliated and twisted, it is due to erosion during the oxidation of MWCNTs with H_2_O_2_ as shown in (Figure 1f). The oxidizing agent causes extreme etching on graphitic surface of MWCNTs. This observation rhymes well with the afore-mentioned outcomes of Raman spectroscopy are based on preferential oxidation at defect sites and disintegration of nanotubes. Nevertheless, the wall structures of the MWCNTs were partially eroded by oxidation within 24 h; however, rest of the fragments possessed better graphitic structure [49].

### 3.3. Thermal Conductivity of MgO-Filled Silicone Rubber

**Figure 5:**
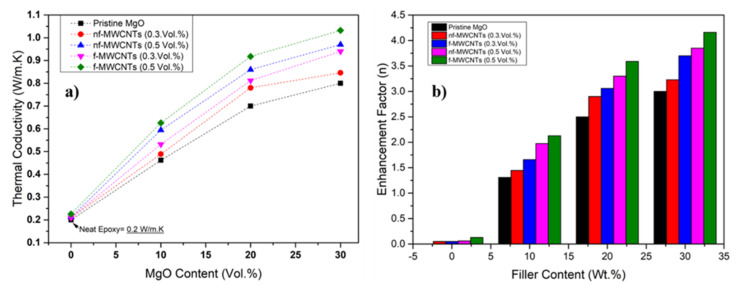
(**a**) Thermal conductivity of MgO/P-MWCNTs and MgO/H_2_O_2_-MWCNTs/silicone elastomer composites; (**b**) Enhancement in thermal conductivity as a function of filler content.

Thermal conductivity of elastomer composite was carried out with advance laser flash operating system (NETZSCH LFA 457 MicroFlash Jeonju, South Korea). Equipped with an infrared detector on top LFA is a contact-less transient thermal analysis technique, extensively utilized for a broad variety of materials ranging between 0.01 mm^2^/s to 1000 mm^2^/s. It works with combination of latest technology with state-of-the-art data analyzing methods, that are completely automated techniques where optimal signal-to-noise ratio is achieved. Both finite pulse effect and heat loss are taken into consideration for processing of acquired data in this technique [50]. This LFA technique has a clear advantage over hot disk method and transient plane-source (TPS) thermal conductivity analyzing techniques. Having discrepancies between heat transfer process and data analysis used by idealized models, the hot disk and transient plane-source TPS methods suffer considerable systematic flaws when applied to lower thermal conductivity/thermal insulation materials [51]. Thermal conductivity is shown in Figure 5 for different loading of MgO and MgO-MWCNT into silicone elastomer. The thermal conductivity of pristine silicone elastomer is 0.2 W/m·K whereas, that of MgO/silicone elastomer composites increases along MgO content loading. The highest total MgO filler content considered in this work was 30 vol% because higher percentages are known to compromise rheological control [22]. The MgO-incorporated silicone rubber exhibits greater thermal conductivity due to greater intrinsic thermal conductivity (Figure 5a). Although, at a lower concentration, the stuffed silicone rubber demonstrates relatively low thermal conductivity as compared to what is expected, probably due to the failure of the nanoparticles to develop perfect thermally conductive pathways lower than 10 vol% loading. However, the nanoparticles, the superfine size, and greater surface energy enable them to become hard to scatter uniformly in silicone rubber. Consequently, most of the nanoparticles get disintegrated through silicon rubber and are unable to produce conductive pathways in the matrix. At 20 vol% concentration or above, nanoparticles started to develop a dense structure inside the matrix, that is more firm and thicker that provides heat conductive pathways due to huge volume proportion of nanoparticles at identical mass fraction. It can be seen, from FE-SEM micrographs, that the nano-sized MgO particles are not distributed uniformly in the elastomer matrix as in Figure 1g,h. However, with an increase in filler loading, MgO nanoparticles contact more. The condition of filler diffusion is crucial, filler units must be cohesive to develop a continuous heat conduction path and increase the thermal properties positively [52]. However, the composite shows greater thermal conductivity. The thermal conductivity is enhanced 40-fold (1.032 W/m·K) in comparison to neat elastomer with the addition of 30 vol% MgO loading filled with 0.5 vol% H_2_O_2_-MWCNTs (Figure 5b). The application of MWCNTs inter-connective paths between MgO and the matrix. FESEM analysis of the composites displayed a non-homogenous distribution at higher loading of MWCNTs Figure 1g,h. On the other hand, the existence of interfacial associations among the nanotubes, MgO, and the matrix is important in creating thermal conductive pathways, is not obvious. The higher increase of thermal conductivity of H_2_O_2_-MWCNTs compared to pristine MWCNTs is due to the functionalization that provides activation of organic groups at the MWCNTs surface [53]. In the course of processing, carbon nanotubes were properly distributed in the composite with low MgO percentage loading—i.e., 10 and 20 vol%—but re-agglomerated after incorporation with higher loading thus being entrapped between elastomer molecules. Subsequently, a separation between the CNTs and MgO ensues due to inhomogeneous mixing. This separation inhibits heat flow from particle to particle, leading to the rapid decrease of thermal conductivity at filler loading with 30 vol% MgO [54]. The thermal conductivity of 20 vol% hybrid composite with 0.5 vol% H_2_O_2_-MWCNTs content is 1.006 W/m·K which is a 44% increase paralleled to the 31% increase in MgO/pristine MWCNTs (0.918 W/m·K). Composites with H_2_O_2_ treated MWCNTs show higher thermal conductivity than those with pristine MWCNTs because the MWCNT surfaces are altered; creating good cohesion between filler and silicone resins matrix in composites will be signified (Figure 5b). Better adhesion between filler and polymer matrix can decrease the thermal blockade of the interface of filler and matrix [54].

## 4. Conclusions

This work presents a simple method to increase the thermal conductivity of silicone elastomer by using a low amount of high-aspect-ratio MWCNTs in MgO-filled silicone rubber in an attempt to find a suitable and convenient method for industrial application. The thermal conductivity is linearly increased with the addition of MgO nanoparticles. For improving the heat transfer in composites between the matrix and fillers, the high- aspect-ratio MWCNTs were incorporated in the elastomer matrix after functionalization with H_2_O_2._ Functionalized MWCNTs were used as source material before their incorporation into the MgO polymer matrix. The functional group residues suggest the effects of particle dispersion on the nanotubes in the matrix at various MgO loading. The thermal conductivity is enhanced 40-fold (1.032 W/m·K) in comparison to neat elastomer with the addition of 30 vol% MgO loading filled with 0.5 vol% H_2_O_2_-MWCNTs. The developed composite with low filler content will be appropriate for heat dissipation applications.

## Data Availability

Not Applicable.
